# Application of Autochthonous Biofilm-Forming *Enterococcus hirae* Kr8 Strain in Relation with Enterocin M in Broiler Rabbits and Their Effect on the Rabbit Meat Quality: Risk or Protection?

**DOI:** 10.1007/s12602-023-10097-z

**Published:** 2023-06-06

**Authors:** Monika Pogány Simonová, Ľubica Chrastinová, Eva Bino, Anna Kandričáková, Zuzana Formelová, Andrea Lauková

**Affiliations:** 1 Centre of Biosciences of the Slovak Academy of Sciences, Institute of Animal Physiology, Šoltésovej 4-6, 04001 Košice, Slovakia; 2 Department of Animal Nutrition, National Agricultural and Food Centre, Hlohovecká 2, 95141 Nitra-Lužianky, Slovakia

**Keywords:** Enterocin, *Enterococcus hirae*, Meat quality, Broiler rabbits, Essential amino acids

## Abstract

Around weaning, rabbits are sensitive to gastrointestinal diseases, mostly of bacterial origin, including enterococci (*Enterococcus hirae*), clostridia, and coliforms. Preventive use of postbiotics—enterocins—as feed additives can reduce this problem. Therefore, simulation of spoilage/pathogenic environment applying the autochthonous, biofilm-forming *E. hirae* Kr8^+^ strain in rabbits and its influence on rabbit meat quality as well as the protective effect of Ent M on rabbit meat properties and quality in infected animals was tested. Ninety-six rabbits aged 35 days, both sexes, meat line M91 breed were divided into one control (CG) and three experimental (EG1, EG2, and EG3) groups. The rabbits in CG received standard diet, without any additives, rabbits in EG1 received 10^8^ CFU/mL of Kr8^+^ strain (at a dose of 500 μL/animal/day), to rabbits in EG2 the Ent M (50 μL/animal/day), and in EG3, combination of the Kr8^+^ and Ent M was applied in their drinking water during 21 days. The experiment lasted 42 days. The Kr8^+^ strain did not attack the gastrointestinal tract and have any adverse effect on the meat quality of rabbits. Moreover, improved weight gains, carcass parameters, and higher essential fatty acid (EAA) and amino acid (EAA) content of rabbit meat point rather to its possible beneficial potential in rabbit nutrition. Administration of Ent M improved most of the tested parameters: animal weight and meat physicochemical and nutritional properties, with a focus on EFA and EAA. During combination of both additives, their synergistic impact was noted, improving the nutritional quality, mostly the EAA content of rabbit meat.

## Introduction

Rabbit meat is a healthy, delicious meat, easy to cook and adaptable to diets of children, elderly, and cardiovascular patients. It is lean meat with low-fat and cholesterol content, providing excellent nutritional quality due to its high biological protein value, involving all essential amino acids (AAs), especially threonine and lysine as well as polyunsaturated fatty acids (PUFA). Rabbit meat also contains a high level of potassium, phosphorus, and selenium. It is the richest source of vitamin B12, but low in sodium, therefore can prevent pernicious anemia and high blood pressure [1, 2]. Fortification of rabbit diet with natural bioactive compounds—pre-, pro-, syn-, and postbiotics, organic and fatty acids, antioxidants, herbal extracts, and phytoadditives—helps to obtain rabbit meat considered as functional food [2], improving its quality and nutritional properties. These bioactive substances are usually used with preventive aim to stabilize/improve the health status and productivity of animals, but they can have also medicinal/protective effect on animals’ performance during disbalances of nutritional, environmental, and physiological origin.

Various enteropathies of multifactorial etiology, mostly of dietetic, bacterial, and/or viral origin, especially around the weaning period can cause digestive disturbances, reflected in impaired health status, higher morbidity and mortality, and reduced productivity of rabbits. Even colibacillosis and clostridiosis are the most frequent gastrointestinal diseases detected in rabbit farms [3, 4]; staphylococci and enterococci are also able to avoid intestinal infections, usually in synergism with other pathogens [5, 6]. *Enterococcus hirae* are usually commensal bacteria of animal gut, but they have been also associated with septicaemia and acute enteropathies, particularly in poultry [7], and already isolated also from suckling rabbits with diarrhea [8]. Most of these bacteria possess several virulence factors, such as the biofilm-forming ability and antibiotic resistance, which are usually encoded by genes, leading to inadequate treatment with commonly used antibiotics and ineffective elimination of these bacteria.

In recent years, bacteriocins—natural antimicrobial compounds of proteinaceous character produced mostly by lactic acid bacteria [9]—represent a new promising approach in animal feeding due to their potential safety, health benefits, and improved product quality compared to synthetic preservatives. These postbiotics, involving commercial nisin and gallidermin and also novel, noncommercial enterocins (Ent; bacteriocins produced by enterococci, such as Ent 2019, Ent 4231, Ent A/P, Ent M, durancin ED26E/7), have already been used in rabbit nutrition with beneficial effect [10-15]. Ent M is a novel enterocin (variant of Ent P) produced by *E. faecium* AL41 strain (a strain isolated from animal waste in our laboratory and deposited as CCM8558 in the Czech Culture Collection in Brno, Czech Republic), which was purified to homogeneity and tested under in vitro conditions [16] and experimentally applied to rabbits and horses with beneficial effects on growth performance, intestinal microbiota, and immune status of the animals, as well as on nutritional quality of rabbit meat [10, 17, 18].

In addition to the general benefits of Ent M in rabbits already described, we decided to know how this natural additive can protect rabbit meat during intestinal bacterial disorders. Therefore, the aim of this study was to simulate the spoilage/pathogenic environment applying a biofilm-forming autochthonous strain *E. hirae* Kr8^+^ [19] in rabbits and to test its influence on rabbit meat quality on the one hand and to investigate the protective effect of Ent M on the characteristics and quality of rabbit meat in infected animals on the other hand.

## Material and Methods

### Animals, Housing, and Experimental Design

The experiment was carried out in cooperation with the National Agricultural and Food Centre (NAFC) in Nitra-Lužianky (Slovakia). All animal care and experimental procedures followed the guidelines stated in the Guide for the Care and Use of Laboratory Animals approved by the Slovakian State Veterinary and Food Administration of the Slovak Republic and the ethics committees of both institutions (permission code: SK CH 17016 and SK U 18016).

Ninety-six rabbits weaned at five weeks of age (meat line M91; both sexes, equal ratio of males to females per treatment) were divided into one control (CG) and three experimental groups (EG1, EG2, and EG3), 24 animals in each group. Rabbits were kept in standard cages (type D-KV-72, 61 × 34 × 33 cm, supplied by Kovobel company, Domažlice, Czech Republic), two animals per cage. A cycle of 16 h of light and 8 h of darkness was used during the experiment. Temperature (16 ± 4 °C) and humidity (70 ± 5%) were maintained throughout the experiment using heating and ventilation systems, and data were continuously recorded using a digital thermograph placed at the same level as the cages. Rabbits were fed a commercial diet for growing rabbits (Table 1), with ad libitum access to water throughout the experiment. The mean live weight of the rabbits at the beginning of the experiment was 1035.8 ± 105.0 g (Table 2). Rabbits in the EG1 group received the biofilm-forming *Enterococcus hirae* Kr8^+^ strain (at a concentration of 1.0 × 10^8^ CFU/mL) in drinking water at a dose of 500 μL/animal/day for 21 days. The strain was marked with rifampicin to differ it from total enterococci and prepared as previously described by Strompfová et al. [20]. Animals in the EG2 group were treated with Ent M (prepared according to Mareková et al. [16]), at a dose of 50 μL/animal/day, with an activity of 12,800 AU/mL, in a concentration of 0.8 g/L, and rabbits in the EG3 group were administered with a combination of both additives, *E. hirae* Kr8^+^ and Ent M for 21 days. Activity of Ent M was tested by the agar spot test according to De Vuyst et al. [21] against the main indicator strain *E. avium* EA5 (isolate from piglet feces, our laboratory). The doses of the additives and the method of their application were decided on the basis of our previous in vitro studies testing the inhibitory activity of Ent M against target bacteria and an experiment with bacteriocin-producing *E. faecium* CCM7420 strain derived from rabbits [22]. Based on our previous experiments, in which these additives can be dissolved in distilled water and/or phosphate buffer [16], the additives were first applied to 100 mL of drinking water in all cages, and after consuming this volume, the rabbits had access to water ad libitum. Control rabbits (CG group) had the same conditions but without application of additives to the drinking water and were fed a commercial diet. Drinking water was supplied via nipple drinkers. The experiment lasted 42 days.

**Table 1:** Ingredients and composition of the commercial diet

Feed ingredients (%)	Diet	Chemical analysis, minerals, and vitamins (g^a^, mg^b^/kg feed)	Diet
Dehydrated lucerne meal	36.0	Dry matter^a^	900.98
Extracted sunflower meal	5.5	Crude protein^a^	176.61
Oats	13.0	Crude fiber^a^	177.84
Wheat bran	9.0	Fat^a^	33.07
Dry malting sprouts	15.0	Ash^a^	69.20
Extracted rapeseed meal	5.5	Nitrogen-free extract^a^	444.25
Barley	8.0	Organic compounds^a^	831.78
DDGS	5.0	Acid detergent fiber (ADF)^a^	208.10
Sodium chloride	0.3	Neutral detergent fiber (NDF)^a^	352.89
Premix minerals^*^	1.7	Hemicellulose^a^	144.79
Limestone	1.0	Cellulose^a^	163.16
		Starch^a^	133.06
		Calcium^a^	9.22
		Phosphorus^a^	5.25
		Magnesium^a^	2.53
		Sodium^a^	1.46
		Potassium^a^	13.84
		Iron^b^	355.26
		Zinc^b^	142.08
		Copper^b^	20.43
		Metabolic energy (MJ/kg)	11.0

*DDGS* dried distillers grains with solubles

*Premix contains per kg: calcium 6.73 g; phosphorous 4.13 g; magnesium 1.90 g; sodium 1.36 g; potassium 11.21 g; iron 0.36 g; zinc 0.13 g; copper 0.03 g; selenium 0.2 mg; vitamin mixture provided per kg of diet: vitamin A 1,500,000 IU; vitamin D3 125,000 IU; vitamin E 5000 mg; vitamin B1 100 mg; vitamin B2 500 mg; vitamin B6 200 mg; vitamin B12 0.01 mg; vitamin K3 0.5 mg; biotin 10 mg; folic acid 25 mg; nicotinic acid 4000 mg; choline chloride 100,000 mg

^a^g/kg

^b^mg/kg

**Table 2:** The effect of *E. hirae* Kr8+ (EG1), Ent M (EG2), and their combined (EG3) application on the growth performance of rabbits

	EG1	EG2	EG3	C	*P* value
Number of rabbits	(*n* = 24)	(*n* = 24)	(*n* = 24)	(*n* = 24)	
Initial live weight (35 d), g	994.2 ± 85.9^a^	1077.1 ± 73.6^b^	1065.4 ± 162.3^ab^	1006.3 ± 98.3^ab^	0.0146
Intermediate live weight (56 d), g	2005.0 ± 182.5^ab^	2070.9 ± 164.8^a^	2030.4 ± 170.3^ab^	1918.3 ± 197.8^b^	0.0302
Final weight (70 d), g	2745.6 ± 271.8	2789.4 ± 234.3	2750.6 ± 163.7	2630.0 ± 226.1	0.1487
Average daily gain (g d^−1^)	41.07^a^	40.77^b^	39.65^c^	38.66^da^	< 0.0001
Feed conversation ratio between 35 and 56 days of age (g/g)	3.16^a^	2.94^b^	2.97^c^	3.03^d^	< 0.0001
Feed conversation ratio between 56 and 77 days of age (g/g)	4.44^a^	4.15^b^	4.34^c^	4.29^d^	< 0.0001
Feed conversation ratio per kg gain	3.64^a^	3.32^b^	3.51^c^	3.57^d^	< 0.0001
Mortality (*n*)	2	4	3	3	

35–56 days of age—application of *E. hirae* Kr8+ strain, Ent M, and their combination

56–70 days of age—after the tested additives cessation

^a,b,c,d^Mean values marked with different letters differ significantly at *P* ≤ 0.05

### Performance Traits, Slaughtering, and Sampling

Body weight and feed consumption were measured weekly during the experiment; average daily weight gain (ADWG) and feed conversion were calculated mathematically. Mortality and morbidity were also recorded daily in all groups.

On days 21 and 42, eight animals from each group were randomly selected for slaughter; they were stunned with electronarcosis (50 Hz, 0.3 A/rabbit/4 s) in an experimental slaughterhouse, immediately hung by their hind legs on a processing line, and quickly bled by cutting the jugular veins and carotid arteries. After bleeding, the *longissimus thoracis and lumborum* (LTL) muscles were separated by removing skin, fat, and connective tissue, cooled, and stored for 24 h at 4 °C until physicochemical analysis was initiated.

### Physicochemical, Amino Acid, and Fatty Acid Analysis

Ultimate pH was determined 24 h *postmortem* (p.m.) using a Radelkis OP-109 (Jenway, Essex, England) with a combined electrode penetrating 3 mm into the LTL. Electrical conductivity (μS·cm^−1^) defined as muscle locations was evaluated using PMV 51 (Tecpro Metall GmbH, Neuss, Germany). Color measurements were performed on LTL surface of the carcass 24 h after bleeding. Color characteristics were expressed using the CIE L*a*b system (lightness—L*, 0: black and 100: white) (redness and greenness—a*; yellowness and blueness—b*) using a Lab Miniscan (HunterLab, Reston, VA, USA). Lightness measurements were also made at room temperature. Total water, protein, and fat contents were determined using an INFRATEC 1265 spectroscope (FOSS, Tecator AB, Höganäs, Sweden) and expressed in g/100 g. The principle of near-infrared transmission (NIT) is based on the fact that the measured sample absorbs the near-infrared light at different wavelengths according to different characteristics such as fat or protein content [23]. From these values, the energy value was calculated [EC (kJ/100 g) = 16.75 × protein content (g/100 g) + 37.68 × fat content (g/100 g)] [24]. Water holding capacity (WHC) was determined by the compression method at constant pressure [25]. The analyzed samples (weighing 0.3 g) were placed on filter papers (Schleicher and Schuell No. 2040B, Dassel, Germany) with previously weighed tweezers. Together with the papers, the samples were placed between Plexiglas plates and then subjected to a pressure of 5 kg for 5 min. The results were calculated from the difference in weight of the slips with aspirating spot and the clean filter paper.

The FA composition of the LTL samples was determined by the method of Ouhayoun [26] by gas chromatography of fatty acid methyl ester (FAME) on a GC 6890N (Agilent Technologies (Schweiz) AG, Basel, Switzerland). Results were expressed as percentages of total FAs. FA composition varied widely and was expressed as the proportion of saturated fatty acids (SFA), monounsaturated fatty acids (MUFA), polyunsaturated fatty acids (PUFA), and n6/n3 index.

AA levels were measured in fat-free samples by ion-exchange chromatography (free AA) and liquid chromatography (total AA) after acid hydrolysis in 6 M HCl and methionine and cystine (sulfur AA) after oxidation hydrolysis, with hydrogen peroxide and formic acid. An AAA 400 analyzer (INGOS s.r.o., Prague, Czech Republic) was used for AA separation.

### Statistical Analysis

Treatment effects on tested parameters were analyzed using one-way analysis of variance (ANOVA) with Tukey post hoc test. All statistical analyses were performed using GraphPad Prism statistical software (GraphPad Prism version 6.0, GraphPad Software, San Diego, CA, USA). Differences between mean values of different dietary treatments were considered statistically significant at *P* < 0.05. Data are expressed as means and standard deviations of the mean (SD).

## Results

The animals were in good health throughout the experiment (Table 2). Higher ADWG was recorded in all experimental groups compared to control data (*P* < 0.001), surprisingly with the highest level in animals receiving *E. hirae* Kr8^+^ (EG1; by 6.2% compared to C), but also with the highest feed conversion ratio (FCR; *P* < 0.001). On the other hand, the lowest FCR was observed in the EG2 group receiving Ent M (*P* < 0.001).

After administration of the *E. hirae* Kr8^+^ strain, the highest carcass yield (although not significant), lowest electrical conductivity (*P* < 0.05) and lightness (*P* < 0.05), and highest WHC (*P* < 0.001) were noted in the meat samples (Table 3). Only pH (*P* < 0.05) and electrical conductivity (*P* < 0.05) were affected by Ent M dietary supplementation. The most significant changes of meat physicochemical properties were observed in rabbits fed a combination of both supplements simultaneously; the highest preslaughter live weight (*P* < 0.05), bloodless body weight and dressed carcass weight (NS), energy value, fat content (*P* < 0.001), meat color parameters (*P* < 0.05), and the lowest pH (*P* < 0.01) and water content (*P* < 0.001) were determined. In this EG3 group, higher values of carcass yield, meat color parameters, and energy content were determined even 21 days after withdrawal of additives.

**Table 3:** The effect of *E. hirae* Kr8+ (EG1), Ent M (EG2), and their combined (EG3) application on the growth performance of rabbits and on the physicochemical composition of rabbits’ *Longissimus thoracis and lumborum* (LTL; means ± SD)

	EG1	EG2	EG3	C	*P* value
	Day 21				
Live weight before slaughter (g)	2110.0 ± 101.0^ab^	2100.0 ± 29.0^ab^	2138.0 ± 90.0^a^	2044.0 ± 67.0^b^	0.0390
Bloodless body weight (g)	2020.0 ± 94.0	2008.0 ± 45.0	2030.0 ± 92.0	1955.0 ± 65.0	0.2364
Dressed carcass weight (g)	1000.0 ± 41.0	976.0 ± 50.0	1005.0 ± 63.0	976.0 ± 40.0	0.6037
Carcass yield (%)	53.48 ± 1.22	52.82 ± 1.60	53.31 ± 1.48	53.30 ± 1.76	1.0000
pH 24 h after killing	5.82 ± 0.05^ab^	5.77 ± 0.07^a^	5.76 ± 0.05^ab^	5.86 ± 0.04^b^	0.0027
Water content (g/100 g)	76.24 ± 0.45^a^	76.24 ± 0.45^a^	74.53 ± 0.61^b^	76.23 ± 0.61^a^	< 0.0001
Protein content (g/100 g)	23.22 ± 0.30	22.98 ± 0.63	23.21 ± 0.37	23.04 ± 0.40	0.5325
Fat content (g/100 g)	1.29 ± 0.29^a^	1.22 ± 0.18^a^	1.83 ± 0.31^b^	1.22 ± 0.29^a^	< 0.0001
Electrical conductivity (μS)	1.63 ± 0.67^a^	2.57 ± 1.16^b^	1.67 ± 0.18^ab^	1.78 ± 1.03^ab^	0.0304
L* (lightness)	49.13 ± 0.62^a^	50.02 ± 2.27^ab^	52.97 ± 3.36^b^	52.34 ± 3.24^bc^	0.0020
a* (redness)	0.78 ± 0.29^ab^	0.73 ± 0.47^ab^	1.63 ± 1.22^a^	0.62 ± 0.29^b^	0.0236
b* (yellowness)	7.07 ± 0.66^a^	7.39 ± 1.66^a^	8.81 ± 0.63^b^	7.51 ± 0.67^ab^	0.0060
Water holding capacity (g/100 g)	32.18 ± 1.94^a^	31.15 ± 2.86^ab^	29.61 ± 2.72^ab^	28.87 ± 4.78^b^	0.0239
Energy value (kJ/100 g)	437.36 ± 8.53^a^	430.70 ± 8.47^a^	457.63 ± 15.86^b^	431.77 ± 7.26^a^	< 0.0001
	Day 42				
Live weight before slaughter (g)	2788.0 ± 271.0	2660.0 ± 227.0	2693.0 ± 173.0	2671.0 ± 203.0	0.5587
Bloodless body weight (g)	2665.0 ± 237.0	2570.0 ± 227.0	2595.0 ± 169.0	2580.0 ± 196.0	0.7984
Dressed carcass weight (g)	1418.0 ± 97.0	1353.0 ± 143.0	1410.0 ± 114.0	1324.0 ± 93.0	0.3023
Carcass yield (%)	55.88 ± 1.99	56.17 ± 2.17	57.53 ± 1.63	55.62 ± 0.68	0.1425
pH 24 h after killing	5.88 ± 0.04^a^	5.85 ± 0.09^ab^	5.85 ± 0.05^b^	5.86 ± 0.07^a^	0.7722
Water content (g/100 g)	74.73 ± 0.62	74.97 ± 0.48	74.46 ± 0.61	74.68 ± 1.10	0.5969
Protein content (g/100 g)	23.35 ± 0.20	23.25 ± 0.46	23.55 ± 0.44	23.15 ± 0.38	0.2156
Fat content (g/100 g)	1.43 ± 0.34	1.56 ± 0.17	1.55 ± 0.15	1.42 ± 0.31	0.5663
Electrical conductivity (μS)	1.71 ± 0.87	2.45 ± 1.90	2.85 ± 0.64	3.31 ± 1.05	0.0797
L* (lightness)	48.68 ± 2.99^a^	51.03 ± 5.47^ab^	54.41 ± 2.47^b^	52.34 ± 3.91^ab^	0.0444
a* (redness)	0.85 ± 0.22	0.79 ± 0.44	1.40 ± 0.93	1.22 ± 0.58	0.1513
b* (yellowness)	6.27 ± 0.59	6.53 ± 1.02	7.60 ± 1.18	6.48 ± 1.03	0.0487
Water holding capacity (g/100 g)	29.68 ± 6.47	31.38 ± 2.86	28.23 ± 2.96	30.67 ± 4.65	0.5365
Energy value (kJ/100 g)	444.87 ± 15.68	448.04 ± 12.87	452.96 ± 3.93	441.28 ± 7.79	0.1792

^a,b,c^Mean values marked with different letters differ significantly at *P* ≤ 0.05

The FA composition of the LTL muscles is shown in Tables 4 and 5. In intramuscular fat, the highest percentage of MUFA (36.101–40.015%) was determined during additive application, followed by SFAs (36.649–37.074%) and PUFAs (7.162–7.482%; Table 4). The concentration of SFAs also increased compared to the control data. A significant increase in lauric acid (*P* < 0.01) and heptadecanoic acid (*P* < 0.05) was noted in EG2 rabbits receiving Ent M alone, compared to rabbits administered Ent M with Kr8^+^ (EG3). Meat samples from the Ent M-treated groups EG2 and EG3 showed decreased MUFA levels (*P* < 0.001) compared to the Kr8^+^ strain (EG1) and CG, with the lowest value of oleic acid in the EG3 group (*P* < 0.05). PUFA values also decreased in all experimental groups, with the lowest level in EG2 (Ent M; *P* < 0.001). Among the tested PUFAs, arachidonic acid (ARA), eicosapentaenoic acid (EPA), and docosahexaenoic acid (DHA) were noted as the highest in LTL with Ent M supplementation alone (EG2), in contrast to their lowest level after the combination of Ent M and Kr8^+^ strain (EG3). However, the highest level of α-linoleic acid (ALA) was measured in this EG3 group. On the other hand, total EFAs and PUFA/SFA ratio were elevated in all experimental groups compared to control data. The highest *n*-6/*n*-3 ratio was described in the EG3 group (*P* < 0.001). Three weeks after withdrawal of the additives (day 42 of the experiment), most of the tested FA levels tested did not show relevant changes compared to the data obtained during Ent M and Kr8^+^ application (Table 5). While SFAs and MUFAs were detected at lower concentrations in the experimental groups, similarly to day 21, there was a change in PUFA (increase in EG2 vs. EG1, EG3; *P* < 0.001; EG2 vs. C: NS) and total EFA concentrations (increase in EG2 vs. EG1: *P* < 0.001; EG2 vs. EG3: *P* < 0.05). Surprisingly, total EFAs decreased compared to the control data. Interestingly, a reduced *n*-6/*n*-3 ratio (*P* < 0.001) was determined after separate application of Kr8^+^ strain (EG1) and Ent M (EG2), but this ratio significantly increased after their combined addition (EG3; *P* < 0.001).

**Table 4:** The effect of *E. hirae* Kr8+ (EG1), Ent M (EG2), and their combined (EG3) application on the growth performance of rabbits and on the fatty acid content in *Longissimus thoracis and lumborum* (LTL) 24 h *postmortem* (% from ΣFA), 56 days of age

	EG1	EG2	EG3	C	*P* value
	Day 21				
C12:0 (lauric a.)	0.107 ± 0.003^ab^	0.110 ± 0.004^a^	0.103 ± 0.004^b^	0.107 ± 0.004^ab^	0.0094
C14:0 (myristic a.)	1.386 ± 0.034	1.364 ± 0.036	1.395 ± 0.014	1.383 ± 0.031	0.1307
C16:0 (palmitic a.)	24.327 ± 0.154	24.486 ± 0.260	24.553 ± 0.166	24.396 ± 0.131	0.0679
C17:0 (heptadecanoic a.)	0.320 ± 0.027^ab^	0.333 ± 0.028^a^	0.303 ± 0.012^b^	0.302 ± 0.033^b^	0.0140
C18:0 (stearic a.)	10.600 ± 0.200	10.550 ± 0.090	10.720 ± 0.258	10.461 ± 0.207	0.0916
Σ tested SFA	36.740 ± 0.384	36.843 ± 0.656	37.074 ± 1.296	36.649 ± 0.740	0.7082
C18:1n-9c (oleic a.)	34.200 ± 3.337^ab^	31.209 ± 1.749^ab^	30.649 ± 3.835^a^	34.474 ± 2.243^b^	0.0126
C18:1 11c/15t (vaccenic a.)	4.924 ± 0.106	4.902 ± 0.022	4.914 ± 0.163	4.945 ± 0.080	0.9410
C20:1 (eicosenoic a.)	0.582 ± 0.081	0.536 ± 0.049	0.538 ± 0.145	0.596 ± 0.083	0.7466
Σ tested MUFA	39.706 ± 1.358	36.647 ± 1.028	36.101 ± 0.381	40.015 ± 1.042	< 0.0001
C18:2n-6 (linoleic a.)	4.930 ± 0.553	4.683 ± 0.491	5.043 ± 0.999	5.121 ± 0.503	0.5398
C18:2 9c/11t (CLA)	0.131 ± 0.012	0.135 ± 0.011	0.128 ± 0.012	0.137 ± 0.011	0.9182
C18:3n-3 (α-linolenic a., ALA)	0.185 ± 0.011	0.173 ± 0.006	0.189 ± 0.022	0.179 ± 0.011	0.9906
C20:4n-6 (arachidonic a., ARA)	1.808 ± 0.231	1.888 ± 0.105	1.715 ± 0.082	1.765 ± 0.240	0.2340
C20:5n-3 (eicosapentaenoic a., EPA)	0.102 ± 0.013	0.110 ± 0.005	0.094 ± 0.016	0.109 ± 0.012	0.9900
C22:5n-3 (docosapentaenoic a., DPA)	0.132 ± 0.004	0.137 ± 0.011	0.131 ± 0.012	0.138 ± 0.010	0.9988
C22:6n-3 (docosahexaenoic a., DHA)	0.033 ± 0.004	0.037 ± 0.004	0.030 ± 0.004	0.033 ± 0.004	0.9993
Σ tested PUFA	7.321 ± 0.106^ab^	7.162 ± 0.022^a^	7.330 ± 0.163^ab^	7.482 ± 0.080^b^	0.0015
Total EFA	8.855 ± 0.597	8.815 ± 0.266	9.095 ± 1.018	8.779 ± 0.677	0.7311
PUFA/SFA	0.324 ± 0.022	0.340 ± 0.018	0.356 ± 0.016	0.317 ± 0.023	0.8812
*n*-6/*n*-3	14.907 ± 0.002^a^	14.410 ± 0.003^b^	15.221 ± 0.004^c^	15.002 ± 0.004^d^	< 0.0001
Fat content (g/100 g)	1.29 ± 0.29^a^	1.22 ± 0.18^a^	1.83 ± 0.31^b^	1.22 ± 0.29^a^	< 0.0001
Cholesterol (g/100 g)	0.373 ± 0.028	0.345 ± 0.051	0.373 ± 0.017	0.346 ± 0.036	0.9134

*SFA* saturated fatty acids, *MUFA* monounsaturated fatty acids, *PUFA* polyunsaturated fatty acids, *EFA* essential fatty acids

^a,b^Mean values marked with different letters differ significantly at *P* ≤ 0.05

**Table 5:** The effect of *E. hirae* Kr8+ (EG1), Ent M (EG2), and their combined (EG3) application on the growth performance of rabbits and on the fatty acid content in *Longissimus thoracis and lumborum* (LTL) 24 h *postmortem* (% from ΣFA), 77 days of age

	EG1	EG2	EG3	C	*P* value
	Day 42				
C12:0 (lauric a.)	0.102 ± 0.003	0.104 ± 0.006	0.102 ± 0.003	0.105 ± 0.004	0.9999
C14:0 (myristic a.)	1.354 ± 0.018	1.342 ± 0.057	1.334 ± 0.028	1.357 ± 0.038	0.9717
C16:0 (palmitic a.)	24.410 ± 0.137	24.357 ± 0.246	24.314 ± 0.218	24.388 ± 0.209	0.8059
C17:0 (heptadecanoic a.)	0.294 ± 0.027	0.329 ± 0.005	0.320 ± 0.045	0.313 ± 0.028	0.9257
C18:0 (stearic a.)	10.439 ± 0.332	10.778 ± 0.131	10.648 ± 0.329	10.749 ± 0.208	0.0645
Σ tested SFA	36.599 ± 0.384	36.910 ± 0.656	36.718 ± 1.296	36.912 ± 0.740	0.8048
C18:1n-9c (oleic a.)	35.162 ± 2.839	35.936 ± 1.612	35.871 ± 2.888	37.267 ± 1.109	0.2951
C18:1 11c/15t (vaccenic a.)	4.951 ± 0.057	4.816 ± 0.116	4.820 ± 0.110	4.854 ± 0.101	0.1788
C20:1 (eicosenoic a.)	0.629 ± 0.079	0.602 ± 0.119	0.574 ± 0.068	0.545 ± 0.074	0.6126
Σ tested MUFA	40.742 ± 1.358^a^	41.354 ± 1.028^a^	41.265 ± 0.381^a^	42.666 ± 1.042^b^	0.0012
C18:2n-6 (linoleic a.)	5.113 ± 0.477	5.715 ± 0.491	5.370 ± 1.041	5.871 ± 0.756	0.1787
C18:2 9c/11t (CLA)	0.142 ± 0.013	0.137 ± 0.009	0.133 ± 0.008	0.139 ± 0.008	1.0000
C18:3n-3 (α-linolenic a., ALA)	0.205 ± 0.011	0.217 ± 0.014	0.217 ± 0.014	0.196 ± 0.014	0.9994
C20:4n-6 (arachidonic a., ARA)	1.995 ± 0.044^ab^	2.056 ± 0.086^a^	1.851 ± 0.212^b^	1.842 ± 0.150^b^	0.0087
C20:5n-3 (eicosapentaenoic a., EPA)	0.119 ± 0.014	0.109 ± 0.010	0.109 ± 0.012	0.111 ± 0.009	0.9923
C22:5n-3 (docosapentaenoic a., DPA)	0.130 ± 0.012	0.145 ± 0.003	0.132 ± 0.010	0.138 ± 0.005	0.9784
C22:6n-3 (docosahexaenoic a., DHA)	0.032 ± 0.003	0.039 ± 0.005	0.034 ± 0.002	0.034 ± 0.003	0.9980
Σ tested PUFA	7.736 ± 0.106^a^	8.418 ± 0.122^b^	7.846 ± 0.163^a^	8.331 ± 0.080^b^	< 0.0001
Total EFA	8.303 ± 0.367^a^	9.743 ± 0.335^b^	8.653 ± 1.472^a^	8.886 ± 0.961^ab^	0.0071
PUFA/SFA	0.323 ± 0.023	0.311 ± 0.019	0.307 ± 0.020	0.299 ± 0.017	0.9386
*n*-6/*n*-3	14.626 ± 0.005^a^	15.237 ± 0.006^b^	14.677 ± 0.010^c^	16.102 ± 0.004^d^	< 0.0001
Fat content (g/100 g)	1.43 ± 0.34	1.56 ± 0.17	1.55 ± 0.15	1.42 ± 0.31	0.5663
Cholesterol (g/100 g)	0.353 ± 0.059	0.323 ± 0.079	0.363 ± 0.046	0.369 ± 0.041	0.7619

*SFA* saturated fatty acids, *MUFA* monounsaturated fatty acids, *PUFA* polyunsaturated fatty acids, *EFA* essential fatty acids

^a,b^Mean values marked with different letters differ significantly at *P* ≤ 0.05

EAA values increased during additive application compared to control data (EG1 vs. C: *P* < 0.001; EG2 vs. C: *P* < 0.01; EG3 vs. C: *P* < 0.05; Table 6). The most significant increase and the highest levels of all EAAs, except valin, were determined in rabbits receiving the combination of Ent M and Kr8^+^ (EG3). This trend was maintained in samples from rabbits administering Ent M alone (EG2) and in combination with Kr8^+^ (EG3) even 3 weeks after cessation of additives, with a significant increase in leucine (EG2 vs. C: *P* < 0.01; EG3 vs. C: *P* < 0.0001). Among all groups, the lowest levels were determined in EG1 (Kr8^+^), except for leucine (EG1 vs. C: *P* < 0.05), which showed the highest value.

**Table 6:** The content of essential amino acids *Longissimus thoracis and lumborum* (LTL) of rabbits (g/100 g)

	EG1	EG2	EG3	C	*P* value
	Day 21				
Total protein (g/100 g)	23.22 ± 0.30^a^	22.98 ± 0.63^b^	23.21 ± 0.37^ab^	23.04 ± 0.40^a^	0.5325
Arginine	1.089 ± 0.059^ab^	1.111 ± 0.056^ab^	1.213 ± 0.152^a^	1.000 ± 0.103^b^	0.0045
Cysteine	0.233 ± 0.020	0.224 ± 0.006	0.249 ± 0.024	0.218 ± 0.014	0.6845
Phenylalanine	0.706 ± 0.038^ab^	0.719 ± 0.029^ab^	0.778 ± 0.097^a^	0.654 ± 0.062^b^	0.0049
Histidine	0.720 ± 0.055	0.742 ± 0.020	0.774 ± 0.126	0.674 ± 0.078	0.0854
Isoleucine	0.661 ± 0.037^ab^	0.676 ± 0.034^ab^	0.736 ± 0.096^a^	0.603 ± 0.066^b^	0.0029
Leucine	1.350 ± 0.078^a^	1.367 ± 0.060^ab^	1.489 ± 0.118^b^	1.242 ± 0.122^a^	0.0004
Lysine	1.456 ± 0.080^ab^	1.486 ± 0.073^ab^	1.621 ± 0.205^a^	1.334 ± 0.138^b^	0.0025
Methionine	0.541 ± 0.039	0.549 ± 0.017	0.593 ± 0.068	0.505 ± 0.044	0.2023
Threonine	0.759 ± 0.038	0.753 ± 0.030	0.817 ± 0.090	0.710 ± 0.058	0.1231
Valin	0.794 ± 0.022	0.816 ± 0.018	0.783 ± 0.091	0.763 ± 0.050	0.6485
Essential amino acids	8.307 ± 0.457^a^	8.444 ± 0.663^a^	9.107 ± 0.124^b^	7.704 ± 0.728^c^	< 0.0001
	Day 42				
Total protein (g/100 g)	23.35 ± 0.20	23.25 ± 0.46	23.55 ± 0.44	23.15 ± 0.38	0.2156
Arginine	1.076 ± 0.115	1.104 ± 0.143	1.144 ± 0.087	1.076 ± 0.111	0.5403
Cysteine	0.241 ± 0.023	0.256 ± 0.041	0.258 ± 0.020	0.250 ± 0.023	0.6243
Phenylalanine	0.699 ± 0.070	0.718 ± 0.089	0.743 ± 0.055	0.704 ± 0.069	0.5263
Histidine	0.709 ± 0.086	0.764 ± 0.132	0.776 ± 0.069	0.751 ± 0.087	0.4512
Isoleucine	0.653 ± 0.071	0.674 ± 0.092	0.693 ± 0.054	0.653 ± 0.070	0.5605
Leucine	1.346 ± 0.139^a^	1.385 ± 0.184^a^	1.428 ± 0.106^a^	1.135 ± 0.137^b^	0.0006
Lysine	1.436 ± 0.155	1.475 ± 0.196	1.530 ± 0.117	1.439 ± 0.150	0.5049
Methionine	0.538 ± 0.058	0.559 ± 0.076	0.576 ± 0.043	0.555 ± 0.056	0.5740
Threonine	0.774 ± 0.066	0.812 ± 0.101	0.819 ± 0.051	0.780 ± 0.064	0.4442
Valin	0.790 ± 0.052	0.811 ± 0.065	0.828 ± 0.039	0.798 ± 0.052	0.4153
Essential amino acids	8.260 ± 0.830	8.557 ± 1.116	8.794 ± 0.635	8.355 ± 0.814	0.5224

^a,b^Mean values marked with different letters differ significantly at *P* ≤ 0.05

## Discussion and Conclusion

Good animal health was observed during the whole experiment. Regarding the biofilm-forming ability of the Kr8^+^ strain as a virulence factor, we hypothesized a “Kr8^+^ attack” on the health and performance of rabbits and a lower tolerance of the host organism to this strain. However, based on higher weight gains, we can assume slower biofilm formation and/or a stimulated immune response in the hostile environment to defend against the spoilage/pathogen agent [27]. On the other hand, the lowest FCR was noted in the EG2 group that administered Ent M (*P* < 0.001). This finding repeatedly confirms the beneficial impact of enterocins on weight gain and FCR due to more intensive metabolic processes in the caecum of rabbits during enterocin administration [18, 28].

Similar to growth performance of rabbits, carcass traits can also be influenced, mostly improved by natural feed supplements—pro-, parapro-, postbiotics, and phytoadditives [2, 29]. Improvement in carcass parameters during Kr8^+^ strain application is associated with faster growth and higher ADWG of rabbits through stimulated nutrient digestibility. Similar results were reported during dietary administration of Ent M and sage [18] as well as Digestarom^®^ supplementation in weaned rabbits [30].

The pH value (measurement of meat acidity) as a result of muscle energy metabolism—conversion of glycogen to lactic acid (LA)—is important for maintaining the microbial quality of meat, because low pH has a bacteriostatic effect during meat storage and also affects the technological and eating quality of the meat. The pH values measured during this experiment agree with literature data [1]. After the application of both additives, a decreased pH (even nonsignificant in EG1) was noted, similar to our previous results when enterocins and a beneficial strain of *E. durans* strain were administered [18, 31, 32], which may be related to changes in the cecal microbiota and higher cecal LA production (data not shown). Lower pH values in meat samples repeatedly confirm the preventive and beneficial effect of postbiotics—enterocins, as well as no negative impact of the biofilm-forming Kr8^+^ strain on the health and meat quality of rabbits. The pH can also influence the color parameters, WHC, and fat content of meat. A negative correlation of pH with color parameters was found only in rabbits treated with the combination of Kr8^+^ and Ent M (EG3), similar to the experimental application of Ent M and sage [18]. However, the L*a*b* color values of rabbit LTL obtained in this experiment were found outside the reported literature data [1]. Higher measured WHC was negatively correlated with reduced pH, similar to the results after dietary administration of beneficial *E. faecium* EF9a strain [33] and Ent M to rabbits [18]. The pH of rabbit meat (a measurement of specific hydrogen ions) is also closely related to electrical conductivity (EC; a nonspecific measurement of both positive and negative ions as an electrical characteristic of muscle [34]). However, the presence of hydrogen ions constitutes only a small fraction of all measured ion concentrations, which affect the correlation between pH and EC depending on the ratio of hydrogen ions to all measured ions. In this experiment, pH was positively correlated with EC, in contrast to those presented by Chrastinová et al. [31], supplementing rabbits with Ent M and durancin ED26E/7. EC as an indicator of membrane integrity, permeability, and ion transport may be a reliable predictor of WHC [35]. With water losses, increased EC data were measured during administration of Ent M and sage to rabbits [18], in contrast to the results measured in this experiment. A positive correlation between WHC and fat content was noted only in the EG3 samples of rabbits administered a combination of both additives, which also showed the highest energy value among all experimental groups. Based on the presented results, the biofilm-forming Kr8^+^ strain did not negatively affect the physicochemical traits; on the contrary, it improved some of them. We hypothesize that ability to form biofilm, which is usually considered as a negative property of bacteria, was eliminated by the degradation of the main virulence factors/surface proteins—enterococcal surface protein (Esp) and aggregation substance (Agg)—due to gastrointestinal proteolytic enzymes [36].

The quality and nutritional value of meat are influenced by its content of FA, AA, and minerals. In this experiment, lower MUFA and PUFA concentrations and higher SFA concentrations were observed compared to our previous results obtained during Ent M and durancin ED26E/7 application to rabbits [31], and lower values of all tested FA were measured than after Ent M and sage administration [18]. The FA composition of rabbit meat can be easily altered by diet and may help to improve the health and nutritional aspect of rabbit meat as a functional food [2]. The expected negative effect of the Kr8^+^ strain on the FA composition of rabbit meat was not confirmed; the slight increase of all tested SFAs, ALA, ARA, and total EFAs does not demonstrate the pathogenicity of Kr8^+^, but on the contrary, a potential benefit of this strain on the quality of rabbit meat can be assumed. Among the SFAs tested, the most significant changes—increase in lauric and heptadecanoic acids—were observed in rabbits receiving Ent M alone, contrary to previous findings [18]. Lauric acid is usually found in high amounts in coconut and palm kernel oil and is characterized by its easy absorption into the host organism, potentially helping with weight loss. Lauric acid has also strong antimicrobial and antiviral properties due to its immune-enhancing effect as the most inhibitory SFA against harmful organisms (herpes simplex virus, HIV virus, papilloma virus, chlamydia, *Staphylococcus aureus* [37]). The main biological function of heptadecanoic acid, contained mainly in milk fat, is the ability to reduce type 2 diabetes and thereby prevent the risks of cardiovascular diseases [38]*.* The increased levels of lauric and heptadecanoic acids in this experiment represent the beneficial potential of Ent M in improving the quality of rabbit meat in order to enhance its already known dietetic and health-promoting values. However, further studies are necessary to develop and confirm these findings.

EFAs are PUFAs that are not metabolized in the body and are involved in various biological processes as precursors of vitamins, derivates, cofactors, etc. Therefore, they can be classified in two ways—into those EFAs obtained from the diet, because animals/humans cannot synthetize them, and into PUFAs with essential functions. EFAs can be divided into two primary families, n-3 and n-6, with the ability of FAs to interconvert within the family [39]. The most important PUFAs are n-6 linoleic acid (precursor of n-6 PUFAs) and n-3 ALA (precursor of n-3 PUFAs, especially EPA and DHA), also known as EFAs. Several clinical studies have shown that linoleic acid, ALA, EPA, and DHA together protect against coronary heart disease. Because of the rich content of these FAs and the low sodium level, rabbit meat is one of the most commonly recommended meat for patients with cardiovascular diseases. Although lower level of all tested PUFAs was noted in the experimental groups, the increased ALA values after the Kr8^+^ strain administration alone (EG1) and in combination with Ent M (EG3) and the higher ARA content in the EG1 and EG2 groups (Ent M) may indicate a better bioavailability of these FAs. The increased concentrations of ARA and ALA also reflect their better ability to convert to other PUFAs based on host requirements, similar to previous results [18]. ARA is one of the EFAs; it is the predominant n-6 FA, converted to prostaglandins, leukotrienes, and other derivates that play a vital role in cell signaling. ARA can also be converted to the longer PUFA—DPA; this conversion ability was not demonstrated in this study in terms of the higher ARA content and lower DPA content recorded in the experimental groups. The *n*-6/*n*-3 ratio corresponds to the values reported by Dalle Zotte et al. [40], but it was found at a lower level (14.626–16.102), compared to the previous experiment with the inclusion of Ent M and sage in the diet, reaching values 22.26–24.33 for LTL [18]. However, we have only preliminary data regarding the effect of enterocins on the FA composition of rabbit meat, but these results suggest that Ent M can improve the biological value of rabbit meat.

Literature data regarding AA content in rabbit meat are still limited and report the influence of rabbit breeds and/or their crosses, weaning age [1, 41, 42], and several bioactive substances—pro- and postbiotics and herbal extracts [18, 31, 43-46]—on AA composition. In general, the EAA content varied at lower level (7.704–9.107), than that found in previous studies after administration of enterocins to rabbits [18, 31], but was similar to the results presented by Bivolarski [42]. Higher EAA levels were measured in all experimental groups, but the most significant increase was observed in rabbits treated with Ent M alone (arginine, phenylalanine, leucine) and also in combination with Kr8^+^ (arginine, phenylalanine, isoleucine, leucine, lysine). The increased EAA concentrations after Kr8^+^ treatment did not show a negative effect of this biofilm-forming strain on rabbit meat quality, as we expected. Thus, the results show that our assumptions about the virulence and potential risk of the Kr8^+^ strain in the rabbit ecosystem were not confirmed, but on the contrary, the results point to its beneficial effect on animal health and productivity. Moreover, the repeated increase of AA content in meat samples after the addition of Ent M confirms its potential to improve rabbit meat quality.

In conclusion, it seems that the biofilm-forming strain *E. hirae* Kr8^+^ did not present any risk in its application and had no adverse effect on growth, carcass characteristics, and meat quality of rabbits. In addition, positive changes in some tested parameters such as faster growth, higher ADWG, improved carcass parameters, and higher SFA, ARA, ALA, EFA, and EAA contents indicate its beneficial potential and possible future use as feed additive in rabbits. Administration of Ent M improved most of the parameters tested, including growth, FCR, carcass quality, meat physicochemical properties, and FA and AA content, with a focus on EFA and EAA, increasing the nutritional quality of rabbit meat and its aspect to be considered as functional food. The combined application of Ent M and Kr8^+^ strain emphasizes not only the protective effect of Ent M but also represents a possible synergistic effect of both additives. We conclude that the inclusion of Ent M and Kr8^+^ strain in the diet as feed additives could increase the weight gain, carcass weight, and nutritional quality of rabbit meat, especially in terms of EFA and EAA content.

## Data Availability

The datasets used and/or analyzed during the current study are available from the corresponding author upon reasonable request.
